# Supplementary material for: Lesion-Function Analysis from Multimodal Imaging and Normative Brain Atlases for Prediction of Cognitive Deficits in Glioma Patients
Source: Cancers (Basel). 2021 May 14;13(10):2373. doi: 10.3390/cancers13102373 (PMC8156090; doi:10.3390/cancers13102373)
Supplement: Supplementary file 1 [file cancers-13-02373-s001.zip › cancers-1196092-supplementary.pdf]

Supplementary Material: Lesion-function analysis from multimodal imaging and normative brain atlases for prediction of cognitive deficits in glioma patients

Martin Kocher, Christiane Jockwitz, Philipp Lohmann, Gabriele Stoffels, Christian Filss, Felix M. Mottaghy, Maximilian I. Ruge, Carolin Weiss Lucas, Roland Goldbrunner, Nadim J. Shah, Gereon R. Fink, Norbert Galldiks, Karl-Josef Langen, Svenja Caspers

Table S1: Patient characteristics

		n	%
**Gender (**male/female)		73/48	60/40
**Age** (years, upper limit)	25/30/40/50/60/70/80	5/17/28/40/23/8	4/14/23/33/19/7
**Performance Status** (ECOG Scale)	0/1/2/3	58/56/6/1	48/46/5/1
**Tumor Type**			
Glioblastoma, *IDH wildtype/mutant/NOS*		67/10/4	56/8/3
Anaplastic astrocytoma, *IDH wildtype/mutant/NOS*		5/16/ 7	4/13/ 6
Anaplastic Oligodendroglioma, *IDH mutant, 1p/19codel.*		12	10
**Tumor Location**			
Frontal left/right		31/28	26/23
Parietal left /right		7/8	6/7
Temporal left/right		22/16	18/13
Occipital left /right		5/4	4/3
**Primary Treatment** [a]			
Resection + RT + TMZ/CCNU/PC		93	77
Biopsy + RT + TMZ/CCNU/PC		12	10
Resection + RT		6	5
Resection + TMZ		3	2
Resection alone		6	5
Biopsy + RT		1	1
**Total Number of Oncologic Interventions**[b]			
1 / 2/ 3 / 4 / 5 / 6 / 7-9		6/6/79/11/11/5/3	5/5/65/9/9/4/3
**Corticosteroids** no/ yes		91/30	25/75
**Anticonvulsants** no/ yes		49/72	40/60
**Recurrence diagnosed by clinical FET PET** no/ yes		63/58	52/48
**Neurological Symptoms**			
None		40	33
Paresis		29	24
Aphasia		17	14
Fatigue		19	16
Visual Field Defect, Diplopia		12	10
Vertigo, confusion		4	3
**Employed** no/yes		45/76	37/63

		Median/ Mean	Range
**Imaging follow-up interval** (months)		14.4/ 30.4	0.6 – 213.7
**Lesion Volume** (ml)			
Resection Cavity	(n= 90)[c]	20.9/ 35.8	0.3 – 172.5
T2/FLAIR abnormalities	(n=121)[c]	53.4/ 70.1***	3.4 – 252.9
T1-Constrast Enhancing	(n= 95)[c]	8.3/ 17.9	0.01 – 122.9
FET-PET positive	(n= 79)[d]	30.3/ 39.5	2.6 – 227.8
**Radiation Dose** (Gy)		60.0/ 59.3	40.1 - 62.0

ECOG: Eastern Cooperative Oncology Group, IDH: Isocitrate Dehydrogenase, NOS: Not otherwise specified, RT: Radiotherapy, TMZ: Temozolomide, CCNU: Chloretyl-Cyclohexyl-Nitroso-Urea = Lomustin, PC: Procarbacin plus CCNU. [a]received until the date of imaging, [b]including biopsy, resection, radiotherapy series, systemic therapy courses. [c]Number of patients affected, [d]including patients with no recurrence according to clinical PET criteria. *** $p<0.001$, 2-sided Kruskal-Wallis test

Table S2: Correlation analysis for cognitive scores and age, education or total volume of lesion types

Cognitive Function Domain/ Test	Age	Edu-cation	Resect. cavity volume	T2/ FLAIR volume	T1-CE Tumor volume	FET-PET Tumor volume
Attention, Processing Speed (TMT-A)	0.46**	- 0.20*	- 0.19*	0.34**	0.28**	0.27**
Processing Speed/ Executive Function (TMT-B)	0.42**	- 0.33**	- 0.17	0.27**	0.20*	0.21*
Executive Function (TMT-B/A ratio)	0.06	-0.34**	0.05	- 0.05	- 0.11	- 0.08
Language Processing (Number Transcoding)	- 0.26**	0.26**	0.04	- 0.03	0.02	- 0.01
Verbal Working Memory (Digit span forward)	- 0.16	0.23*	0.07	- 0.14	- 0.07	- 0.02
Verbal Working Memory (Digit span backward)	- 0.30**	0.28**	0.16	- 0.16	- 0.12	- 0.07
Visual Working Memory (CBT forward)	- 0.40**	0.24**	0.13	- 0.29**	- 0.18*	- 0.15
Visual Working Memory (CBT backward)	- 0.34**	0.33**	0.13	- 0.20*	- 0.24**	- 0.28**
Verbal Episodic Memory (Word list, immediate recall)	- 0.25**	0.26**	0.18*	- 0.29**	- 0.25**	- 0.24**
Verbal Episodic Memory (Word list, delayed recall)	- 0.38**	0.24**	0.26**	- 0.21*	- 0.19*	- 0.20*

Spearman rank correlation coefficients between age, educational level and total volume of lesion types and neurocognitive test scores in different domains. Increasing age and lesion volumes are expected to increase the time needed for TMT-A/B and decrease the scores for the other tests, while educational levels are assumed to act in the opposite way. Unexpectedly, this opposite pattern was also observed for the total volume of the resection cavities. *$p < 0.05$, **$p < 0.01$ (2-sided); Resect. Cavity: volume of resection cavity; T2/FLAIR: volume of T2/FLAIR image changes; T1-CE: volume of contrast-enhancing tumor in T1-weighted MR-images; FET-PET: volume of FET-PET positive tumor

Table S3: Correlation analysis for cognitive scores and clinical variables

Cognitive Function Domain/Test	Inter val	Surgical Proced.	Radioth. Series	Chemoth. Courses	Total Intervent.	Gender f/m	Anticonv. no/yes	Glioma Grade III vs. IV	Glioma MolType AA-wt/-mut/ OD
Attention, Processing Speed (TMT-A)	- 0.17	- 0.13	0.00	- 0.04	- 0.04	45.1/48.7	47.0/47.5	39.1/51.2*	51.9/40.8/32.9*
Processing Speed/ Executive Function (TMT-B)	- 0.13	- 0.11	-0.04	0.00	- 0.04	110.0/122.6	117.9/117.5	100.3/126.1	126.3/109.4/84.2
Executive Function (TMT-B/A ratio)	0.11	0.00	- 0.02	0.05	- 0.01	2.5/2.6	2.5/2.6	2.6/2.5	2.5/2.7/2.6
Language, Word Fluency (Supermarket)	- 0.02	- 0.02	- 0.02	- 0.09	- 0.06	20.8/19.7	20.7/19.8	21.0/19.7	20.1/19.2/22.1
Language Processing (Number Transcoding)	- 0.06	- 0.06	- 0.05	- 0.08	- 0.08	3.5/3.3	3.3/3.3	3.3/3.3	3.3/3.4/3.6
Verbal Working Memory (Digit span forward)	- 0.04	- 0.04	- 0.08	- 0.03	- 0.05	7.2/7.5	7.5/7.3	6.9/7.6	7.5/7.0/6.9
Verbal Working Memory (Digit span backward)	0.20*	0.16	0.08	0.14	0.15	6.4/6.6	6.6/6.5	7.1/6.2	6.3/6.7/7.3
Visual Working Memory (CBT forward)	- 0.01	- 0.02	- 0.16	- 0.11	- 0.12	6.8/6.4	6.6/6.6	7.1/6.3	6.3/6.9/7.7
Visual Working Memory (CBT backward)	0.03	0.05	0.04	0.02	0.03	4.9/4.8	4.6/4.9	5.3/4.5	4.5/5.2/5.6
Verbal Episodic Memory (Word list, immediate recall)	- 0.06	0.03	- 0.02	- 0.08	- 0.04	12.0/11.6	12.1/11.5	12.0/11.6	11.5/11.8/13.1
Verbal Episodic Memory (Word list, delayed recall)	- 0.02	0.08	0.04	- 0.09	- 0.02	4.6/4.5	4.9/4.2	4.7/4.4	4.1/5.2/5.6

Spearman rank correlation coefficients between therapy interval, number of surgical procedures, number of radiotherapy series, number of chemotherapy courses and total number of oncologic interventions and neurocognitive test scores in different domains; mean neurocognitive scores in groups of differing gender, anticonvulsant use, glioma grade and molecular glioma type (AA-wt: anaplastic astrocytoma/ glioblastoma IDH wild-type, -mut: IDH mutated, OD: anaplastic oligodendroglioma); Mann-Whitney-U and Kruskal-Wallis tests, *$p < 0.05$, (2-sided)
